# Safety and Activity of Programmed Cell Death 1 Versus Programmed Cell Death Ligand 1 Inhibitors for Platinum-Resistant Urothelial Cancer: A Meta-Analysis of Published Clinical Trials

**DOI:** 10.3389/fonc.2021.629646

**Published:** 2021-04-01

**Authors:** Zaishang Li, Xueying Li, Wayne Lam, Yabing Cao, Hui Han, Xueqi Zhang, Jiequn Fang, Kefeng Xiao, Fangjian Zhou

**Affiliations:** ^1^ Department of Urology, Shenzhen People’s Hospital, The Second Clinic Medical College of Jinan University, Shenzhen, China; ^2^ Department of Urology, First Affiliated Hospital of Southern University of Science and Technology, Shenzhen, China; ^3^ Department of Urology, Minimally Invasive Urology of Shenzhen Research and Development Center of Medical Engineering and Technology, Shenzhen, China; ^4^ Department of Oncology, The Seventh Affiliated Hospital of Sun Yat-sen University, Shenzhen, China; ^5^ Division of Urology, Department of Surgery, Queen Mary Hospital, The University of Hong Kong, Hong Kong, Hong Kong; ^6^ Department of Oncology, Hospital Kiang Wu, Macau, Macau; ^7^ Department of Urology, Sun Yat-sen University Cancer Center, Guangzhou, China; ^8^ Department of Urology, State Key Laboratory of Oncology in South China, Guangzhou, China; ^9^ Department of Urology, Collaborative Innovation Center of Cancer Medicine, Guangzhou, China

**Keywords:** immunotherapy, urologic neoplasms, review, programmed cell death 1 receptor, programmed cell death 1 ligand

## Abstract

**Background:**

Programmed death 1/ligand 1 (PD-1/L1) inhibitors have acceptable antitumor activity in patients with platinum-resistant urothelial cancer (UC). However, the reliability and comparability of the antitumor activity, safety profiles and survival outcomes of different immune checkpoint inhibitors are unknown. Our objective was to compare the clinical efficacy and safety of anti–PD-1/PD-L1 therapies in platinum-resistant UC patients.

**Methods:**

We reviewed the published trials from the PubMed, Embase and Cochrane Library databases up to August 2020. A well-designed mirror principle strategy to screen and pair trial characteristics was used to justify indirect comparisons. The primary end point was the objective response rate (ORR). The safety profile and survival outcomes were also evaluated. The restricted mean survival time (RMST) up to 12 months was calculated.

**Results:**

Eight studies including 1,666 advanced or metastatic UC patients (1,021 patients with anti–PD-L1 treatment and 645 patients with anti–PD-1 treatment) met the study criteria. The ORRs of anti–PD-1 and PD-L1 therapy were 22% (95% CI, 18%–25%) and 15% (95% CI, 13%–17%) with all studies combined. The proportions of the treated population with a confirmed objective response (I^2^ = 0; *P* = 0.966; HR, 1.60; 95% CI, 1.23–2.07; *P* < 0.001) and disease control (I^2^ = 30.6%; *P* = 0.229; HR, 1.35; 95% CI, 1.10–1.66; *P* = 0.004) were higher with anti–PD-1 therapy than with anti–PD-L1 therapy. The treatment-related adverse events (AEs) (I^2^ = 78.3%; *P* = 0.003; OR, 1.09; 95% CI, 0.65–1.84; *P* = 0.741) and grade 3–5 treatment-related AEs (I^2^ = 68.5%; *P* = 0.023; OR, 1.69; 95% CI, 0.95–3.01; *P* = 0.074) of anti–PD-1 therapy were comparable to those of anti–PD-L1 therapy. The RMST values at the 12-month follow-up were 9.4 months (95% CI,: 8.8–10.0) for anti–PD-1 therapy and 9.3 months (95% CI, 8.8–9.7) for anti–PD-L1 therapy (z = 0.26, *P* = 0.794). There was no significant difference between patients in the anti–PD-1 and anti–PD-L1 groups (12-month overall survival (OS): 43% versus 42%, *P* = 0.765. I^2^ = 0; *P* = 0.999; HR, 0.95; 95% CI, 0.83–1.09; *P* = 0.474).

**Conclusions:**

The results of our systematic comparison suggest that anti–PD-1 therapy exhibits better antitumor activity than anti–PD-L1 therapy, with comparable safety profiles and survival outcomes. These findings may contribute to enhanced treatment awareness in patients with platinum-resistant UC.

## Introduction

Advanced or metastatic urothelial cancer (UC) patients have a poor prognosis (1, 2), and platinum-based first-line chemotherapy is the standard treatment option for these patients (1, 3–5). However, the median overall survival (OS) of UC patients who benefit from combination chemotherapy regimens is only 14 to 15 months (1). When first-line chemotherapy resistance occurs, other regimens have limited efficacy, and these patients have an OS of approximately 6 months (3, 6, 7).

Immune checkpoint inhibitors including programmed death 1 (PD-1) and programmed death 1 programmed death ligand 1 (PD-L1) treatment represent a breakthrough in the treatment of advanced or metastatic UC (8, 9), and the safety and activity of anti–PD-1/PD-L1 therapy for advanced or metastatic UC patients have been confirmed (1). In a multicenter, phase 3 randomized trial (KEYNOTE-045), pembrolizumab showed encouraging survival benefits over chemotherapy in advanced/metastatic, platinum-refractory UC (10, 11). Atezolizumab was also confirmed to have a clinical benefit, as the survival of the immunotherapy group was higher than that of the chemotherapy group (12–15).

Studies have confirmed that the mechanism divergence in the inhibitory pathway influences the clinical effects of PD-1 and PD-L1 therapy (16–18). However, the differences between anti–PD-1 and anti–PD-L1 in advanced or metastatic UC patients have raised uncertainties. A network meta-analysis that compared the survival of patients with PD-1 versus PD-L1 blockade included only two studies, limiting the amount of data available for analysis (19).

In this meta-analysis, we used a well-designed mirror principle strategy that included screening and pairing trial characteristics to adjust for indirect comparisons (20). The durable response rates, survival, and tolerability of anti–PD-1/PD-L1 therapy in patients with platinum-resistant UC were strictly assessed.

## Methods

### Search Strategy

#### Literature Search Strategy

Following the Preferred Reporting Items for Systematic Review and Meta- Analyses (PRISMA) statement, the strategy of the study was determined in advance and uploaded to the PROSPERO online platform. PubMed, Embase, and the Cochrane Library were searched for studies published up to August 2020. The following medical subject heading (MeSH) terms and their combinations were searched in the [Title/Abstract] field: PD-1, PD-L1, programmed death receptor 1, programmed death receptor ligand 1, immune checkpoint inhibitors, and urothelial carcinoma.

### Inclusion and Exclusion Criteria

Eligible studies included the following: (1) patients with advanced or metastatic UC; (2) anti–PD-1/PD-L1 treatment; (3) patients with platinum-resistant disease; (4) clinical trials (phase I, II or III); (5) ≥20 patients who reported responses; and (6) published in the English language. Studies including anti–PD-/PD-L1 treatment with other immunotherapies, anti–PD-/PD-L1 neoadjuvant treatment, and anti–PD-/PD-L1 as maintenance treatment and retrospective studies were excluded. For duplicate publications, only the most recent and complete publication was included.

### Data Extraction

Xueying Li and Zaishang Li independently extracted and summarized the information. A senior researcher (Hui Han) served as the adjudication author and resolved any disagreements. Disagreements among all authors were resolved *via* discussion. The following information was extracted from the studies: first author, year of publication, phase of trials, National Clinical Trial number, clinical trial name, treatments, number of patients, sex, age, physical condition score, follow-up time, objective response rate (ORR), progression-free survival (PFS), OS, and adverse events (AEs). The level of evidence for the evaluated studies was assessed according to the Grading of Recommendations Assessment, Development and Evaluation (GRADE) system and the Oxford system (21, 22).

### Statistical Analysis

The mirror principle was applied to compare anti–PD-1 and anti–PD-L1 therapies (20). The studies were matched based on characteristics including immunotherapy drugs, therapeutic schedule, clinical trial phase, previous treatments, lines of treatment, PD-L1 expression level, Eastern Cooperative Oncology Group (ECOG) performance status, and sex ratio. Studies lacking a relevant variable, for example, the IMvigor130 trial, were also eligible for this study. Only successfully matched studies were further analyzed.

The primary outcome was the ORR. The secondary outcomes were the disease control rate (DCR), AEs, and OS. The AEs were evaluated according to human body systems. Survival data were reconstructed with Engauge software for direct comparisons (19, 23). Reconstructed survival data meta-analysis methods were used to estimate the restricted mean survival time (RMST) up to 12 months and assessed using the method described by Grambsch and Therneau (24–26). RMST analyses were conducted with R software (survRM2 and metafor packages).

The survival rate of affected patients was expressed as the hazard ratio (HR), and the presence of lymph node metastasis was expressed as an odds ratios (OR) (27). Stata version 12 (Stata Corp, College Station, TX, USA) was used for comparisons of the HR or OR and their 95% confidence intervals (CIs). Specified subgroup analyses were conducted for studies that included immune checkpoint inhibitors and other lines of treatment. Statistical heterogeneity between studies was assessed using the chi-square test with a random-effects model if the *P* value was <0.10; otherwise, a fixed-effects model was used. Sensitivity analyses were performed for high-quality studies. Funnel plots, and Begg’s and Egger’s tests were used to screen for potential publication bias.

## Results

### Study Selection and Characteristics

**Figure 1:**
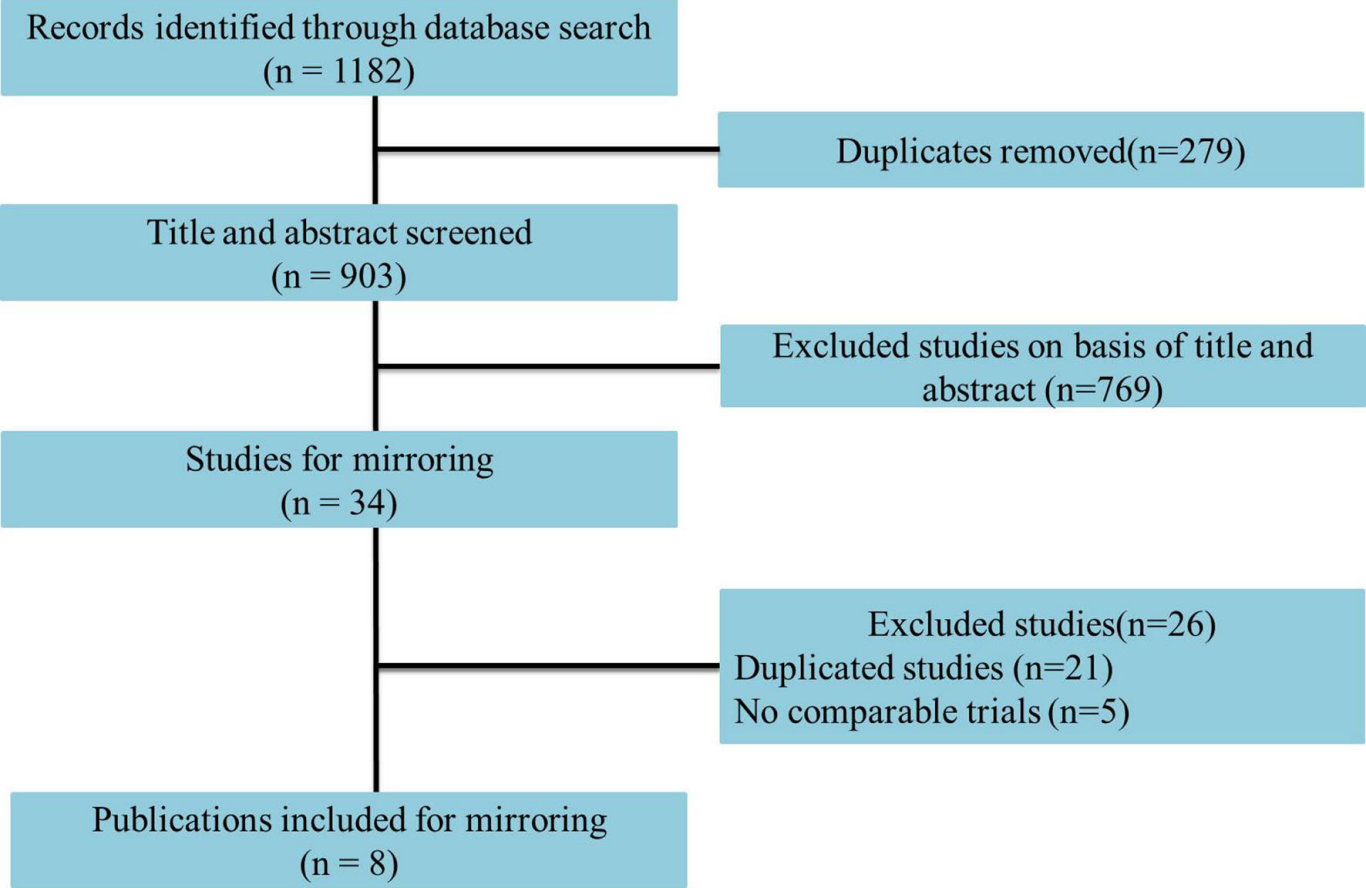
Flowchart of study selection.

Eight studies including 1,666 advanced or metastatic UC patients (1,021 patients with anti–PD-L1 treatment and 645 patients with anti–PD-1 treatment) met the inclusion criteria (**Figure 1**). Detailed characteristics are summarized in **Table 1**. The JAVELIN Solid trial (dose-expansion cohort) (34), in which 90% of patients with ≥1% PD-L1 expression were given avelumab, was matched with the KEYNOTE-012 trial (30). The level of evidence according to the GRADE and Oxford systems for the evaluated studies is shown in **Table 1**. **Table 2** shows the matched outcomes using the mirror principle (32).

**Table 1:** The characteristics of included trials.

Author	Year	NCT Number	RCT name	Clinical Trial Phase	Intervention	Total (n)	Median Age(range)	Male n(%)	ECOGPS=0 n(%)	MPs Median, months (95% CI)	mOS Median, months (95% CI)	Follow-up (month) Median, months (range or IQR)	Level of evidence according to the GRADE system	Level of evidence according to the Oxford System
Fradet et al. (11)	2019	NCT02256436	KEYNOTE-045	III	Pembrolizumab	270	67 (29–88)	200 (74)	119 (44)	2.1 (2.0–2.2)	10.1 (8.0–12.3)	27.7 (median)	Grade 1	1b
Powles et al. (28)	2018	NCT02302807	IMvigor211	III	Atezolizumab	467	67 (43–88)	81 (70)	61 (53)	NA	8.6 (7.8–9.6)	17·3 (range, 0–24·5)	Grade 1	1b
Ohyama et al. (29)	2019	NCT 02387996	CheckMate 275	II	Nivolumab	270	66 (38–90)	211 (78)	145 (54)	1.9 (1.9–2.3)	8.6 (6.1–11.3)	33.7 (minimum)	Grade 2	2b
Rosenberg et al. (30)	2016	NCT02108652	IMvigor210 (Cohort 2)	II	Atezolizumab	310	66 (32–91)	241 (78)	117 (38)	2.7 (2.1–3.9)	7.9 (6.6–9.3)	11.7 (IQR, 11·4–12·2)	Grade 2	2b
Sharma et al. (31)	2016	NCT01928394	CheckMate 032	I/II	Nivolumab	78	66 (31–85)	54 (69)	42 (54)	NA	9.7 (7.3–16.2)	15.2 (IQR, 12·9–16·8)	Grade 2	2b
Powles et al. (32)	2017	NCT01693562	Study 1108	I/II	Durvalumab	191	67 (34–88)	136 (71)	64 (34)	1.5 (1.4–1.9)	18.2 (8.1-NE)	5.8 (range, 0.4–25.9)	Grade 2	2b
Plimack et al. (33)	2017	NCT01848834	KEYNOTE-012	Ib	Pembrolizumab	33	70 (44–85)	23 (70)	9 (27)	2 (2–4)	13 (5–20)	13 (IQR, 5–23)	Grade 2	2b
Apolo et al. (34)	2017	NCT01772004	JAVELIN Solid (dose-expansion cohort)	Ib	Avelumab	44	68 (63–73)	30 (68)	19 (43)	2.9 (1.5–4.4)	13.7 (8.5-NE)	16.5 (IQR, 15.8–16.7)	Grade 2	2b

IQR, interquartile range; HR, hazard ratio; NA, not available, ICI, immune checkpoint inhibitor; Ctl, control.

**Table 2:** The indirect comparison of selected studies based on the mirror principle.

Matched groups	RCT name	Immunotherapy drug	Therapeutic schedule	Clinical trial Phase	Therapeutic schedule	Lines of Treatment	PD-L1 status	ECOG-PS	Male (%) ± 2%
1	KEYNOTE-045	PD-L1	Pembrolizumab	III	Platinum-based chemotherapy	≤3	All	0–1**	72% (+2%)
	IMvigor211	PD-1	Atezolizumab	III	Platinum-based chemotherapy	≤3	All	0–1	72% (−2%)
2	CheckMate 275	PD-L1	Nivolumab	II	Platinum-based chemotherapy	2	All	0–1***	78% (0)
	IMvigor210 (Cohort 2)	PD-1	Atezolizumab	II	Platinum-based chemotherapy	2	All	0–1	78% (0)
3	CheckMate 032	PD-L1	Nivolumab	I/II	Platinum-based chemotherapy	2	All	0–1	70% (−1%)
	Study 1108	PD-1	Durvalumab	I/II	Platinum-based chemotherapy	2	All	0–1	70% (+1%)
4	KEYNOTE-012	PD-L1	Pembrolizumab	Ib	Previous treatment, including platinum-based therapy	≥1	≥1% PD-L1 expression	0–1	69% (+1%)
	JAVELIN Solid (dose-expansion cohort)	PD-1	Avelumab	Ib	Platinum-based chemotherapy	≥1	≥1% PD-L1 expression*	0-1	69% (-1%)

*90% patients with ≥1% PD-L1 expression; **only 2 (0.7%) patients with ECOG performance status of 2; ***only one (0.4%) patient had ECOGperformance status of 3.

The studies included two anti–PD-1 drugs (two studies on pembrolizumab and two studies on nivolumab) and three anti–PD-1 trials (two studies on atezolizumab, one study on avelumab, and one study on durvalumab). The sensitivity analysis, Begg’s test and Egger’s test showed that no bias existed in the selected studies.

### Antitumor Activity

**Figure 2:**
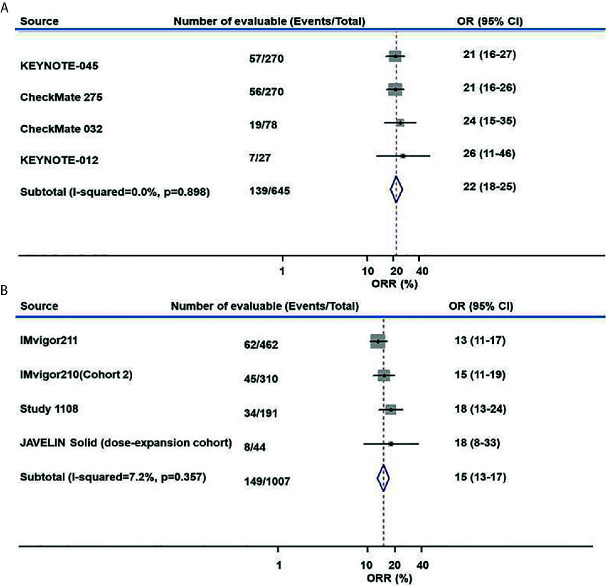
Meta-analysis of pooled odds ratios of an objective response to anti–PD-1 and anti–PD-L1 therapy. **(A)** anti–PD-1, **(B)** anti–PD-L1.

The ORR of anti–PD-L1 therapy was 15% (95% CI, 13%–17%) for all studies combined (**Figure 2A**). The combined ORR of anti–PD-1 therapy was 22% (95% CI, 18%–25%) (**Figure 2B**).

**Figure 3:**
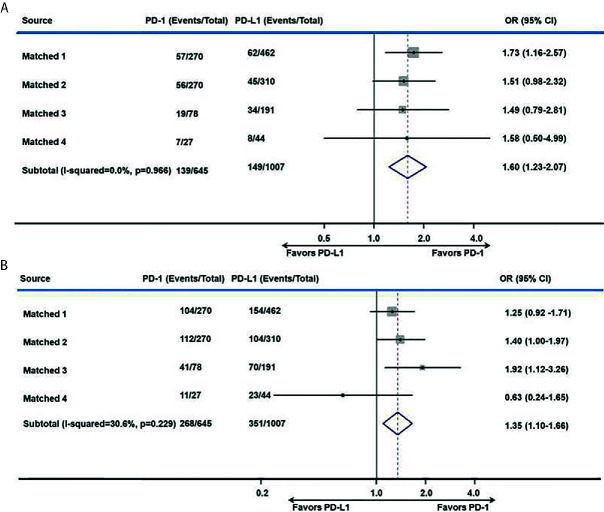
Meta-analysis of pooled odds ratios of a tumor response to anti–PD-1 versus anti–PD-L1 therapy. **(A)** ORR, **(B)** DCR.

After matching, the proportion of the treated population with a confirmed objective response was higher with anti–PD-1 therapy than with anti–PD-L1 therapy (I^2^ = 0; *P* = 0.966; HR, 1.60; 95% CI, 1.23–2.07; *P* < 0.001) among advanced or metastatic UC patients after progression on platinum-based chemotherapy (**Figure 3A**). According to the subgroup analysis, the ORR was also higher with anti–PD-1 therapy than with anti–PD-L1 therapy in studies with a sample size >50 (I^2^ = 0%; *P* = 0.875; HR, 1.60; 95% CI, 1.22–2.08; *P* < 0.001).

The DCR was also higher with anti–PD-1 therapy than with anti–PD-L1 therapy in all included studies (I^2^ = 30.6%; *P* = 0.229; HR, 1.35; 95% CI, 1.10–1.66; *P* = 0.004. **Figure 3B**) and in studies with a sample size >50 (I^2^ = 0%; *P* = 0.404; HR, 1.40; 95% CI, 1.13–1.72; *P* = 0.002).

However, the progressive disease rate of the anti–PD-1 group was comparable to that of the anti–PD-L1 group (I^2^ = 41.7%; *P* = 0.162; HR, 0.82; 95% CI, 0.62–1.10; *P* = 0.189). The investigator-assessed antitumor activity and median duration of response are shown in **Table 3**.

**Table 3:** Adverse events that occurred during the trial period.

Variable	Matched 1		Matched 2		Matched 3		Matched 4	
	anti–PD-L1	anti–PD-1	anti–PD-L1	anti–PD-1	anti–PD-L1	anti–PD-1	anti–PD-L1	anti–PD-1
**Objective response**								
No. objective response (n/N)	62/462	57/270	45/310	56/270	34/191	19/78	8/44	7/27
ORR (%)	13 (11–17)	21 (16–27)	15 (11–19)	21 (16–26)	18 (13–24)	24 (15–35)	18 (8–33)	26 (11–46)
DCR n (%)	154 (33)	104 (39)	104 (34)	112 (42)	70 (37)	41 (53)	23 (52)	11 (41)
CR n (%)	16 (3)	25 (9)	15 (5)	18 (7)	7 (4)	5 (6)	5 (11)	3 (1)
PR n (%)	46 (10)	32 (12)	30 (10)	38 (14)	27 (14)	14 (18)	3 (7)	4 (2)
SD n (%)	92 (20)	47 (17)	59 (19)	56 (21)	36 (19)	22 (28)	15 (34)	4 (2)
PD n (%)	240 (52)	131 (49)	159 (51)	111 (41)	88 (46)	30 (38)	15 (34)	14 (52)
Median duration of response	21.7 (13.0–21.7)	NE (1.6–30.0)	NE (2.0–13.7)	20.3 (11.5-31.3)	NE (0.9-19.9)	9.4 (5.7–12.5)	NE (3-NE)	10 (4–22)
**Adverse events**								
Treatment-related AEs	319 (69)	165 (62)	215 (69)	187 (69)	116 (61)	63 (81)	29 (66)	20 (61)
Treatment-related AEs (3–5 grade)	91 (20)	44 (17)	50 (16)	67 (25)	13 (7)	17 (22)	3 (7)	5 (15)
Treatment-related serious AEs	72 (16)	32 (12)	34 (11)	NA	9 (5)	8 (10)	2 (5)	3 (9)
Treatment-related AEs leading to treatment discontinuation	16 (3)	18 (7)	11 (4)	27 (10)	9 (5)	2 (3)	4 (9)	2 (6)
Treatment-related AEs lead to death	4 (1)	4 (2)	0	3 (1)	2 (1)	1 (1)	0	0
AEs with incidence ≥1%								
Asthenia	51 (43)	17 (5)	21 (8)	19 (6)	0	0	5 (11)	0
Circulatory	0	0	8 (3)	0	3 (2)	0	0	0
Decreased appetite	56 (18)	25 (9)	36 (13)	26 (8)	18 (9)	0	2 (5)	0
Fatigue	116 (25)	37 (14)	93 (34)	52 (17)	37 (19)	28 (36)	9 (20)	6 (18)
Pyrexia	40 (9)	0	28 (10)	17 (5)	15 (8)	0	0	0
Dermatological	120 (26)	84 (32)	54 (20)	120 (39)	33 (17)	39 (50)	8 (18)	2 (6)
Endocrine	53 (45)	19 (5)	0	46 (15)	9 (5)	5 (6)	4 (9)	0
Gastrointestinal	161 (35)	78 (29)	87 (32)	106 (34)	29 (15)	11 (14)	9 (20)	0
Hematogenous	29 (15)	8 (10)	9 (3)	30 (10)	8 (4)	27 (35)	10 (23)	1 (3)
Hepatic	0	0	10 (4)	15 (5)	25 (13)	5 (6)	3 (7)	2 (6)
Renal	0	0	0	5 (2)	1 (1)	1 (1)	0	0
Respiratory	35 (11)	8 (3)	17 (6)	14 (5)	0	7 (9)	1 (2)	0
Others	49 (11)	36 (14)	0	0	11 (6)	10 (13)	0	10 (30)

(1) Circulatory: atrial fibrillation, cardiorespiratory arrest, hypertension, hypotension, myocarditis (2); dermatological: alopecia, dermatitis acneiform, dry mouth, maculopapular, mucosal inflammation, skin reactions, pruritus, rash, stomatitis, tumor flare, uveitis (3); endocrine:adrenal disorder, diabetes, hypothyroidism, hypophysitis, hyperthyroidism, hypersensitivity, hyperglycemia, pituitary disorder, rheumatoid arthritis, thyroid disorder; (4) gastrointestinal: abdominal pain, colitis, constipation, diarrhea, intestinal perforation, increased amylase, nausea, pancreatitis, vomiting (5); hematogenous: anemia, blood alkaline phosphatase increased, creatine phosphokinase, dehydration, hyponatremia, increased blood ALP level, Infusion-related reaction, leukocyte count decreased, lipase elevated, lymphocyte count decreased, neutropenia, thrombocytopenia (6); hepatic: alanine aminotransferase increased, amylase increased, aspartate aminotransferase increased, blood bilirubin increased, hepatitis (7); renal: nephritis, renal failure, urinary tract obstruction (8); respiratory: cough, dyspnea, interstitial lung disease, pneumonitis, respiratory tract infection, respiratory failure, wheezing (9); others: arthralgia, dysgeusia, edema peripheral, muscle spasms, myalgia, myositis, neuromyopathy, pain, paresthesia, peripheral sensory neuropathy, peripheral sensory neuropathy, rhabdomyolysis, toxic encephalopathy.

### Safety Analysis

**Figure 4:**
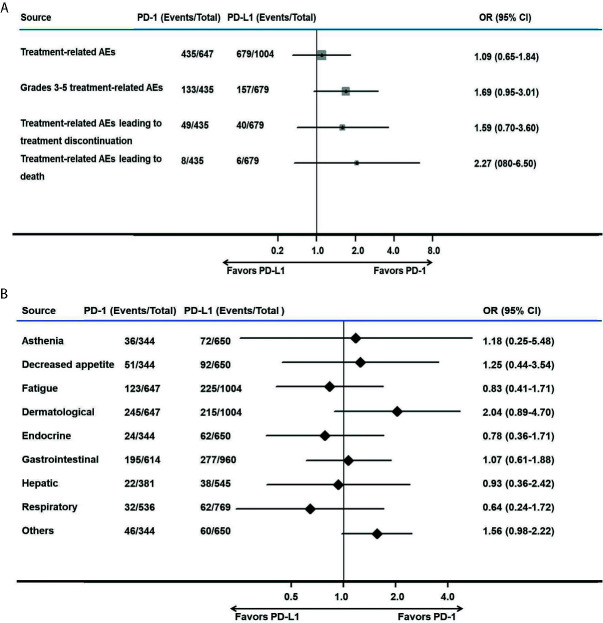
Meta-analysis of pooled odds ratios of adverse events of anti–PD-1 versus anti–PD-L1 therapy. **(A)** adverse events with anti–PD-1 versus anti–PD-L1 therapy, **(B)** adverse events with an incidence ≥1% for anti–PD-1 versus anti–PD-L1 therapy.

The treatment-related AEs (I^2^ = 78.3%; *P* = 0.003; OR, 1.09; 95% CI, 0.65–1.84; *P* = 0.741) and grade 3–5 treatment-related AEs (I^2^ = 68.5%; *P* = 0.023; OR, 1.69; 95% CI, 0.95–3.01; *P* = 0.074) associated with anti–PD-1 therapy were comparable to those of anti–PD-L1 therapy. Various AEs are shown in **Figure 4** and **Table 3**.

No significant difference was found among treatment-related AEs leading to treatment discontinuation (I^2^ = 58.1%; *P* = 0.067; OR, 1.59; 95% CI, 0.70–3.60; *P* = 0.264) and AEs leading to death (I^2^ = 0; *P* = 0.524; OR, 2.27; 95% CI, 0.80–6.50; *P* = 0.127), as shown in **Figure 4A**. Among AEs with an incidence ≥1%, there were no AEs that occurred more frequently in the PD-1 group (**Figure 4B**).

### Survival

The median OS times of patients with anti–PD-L1 therapy and anti–PD-1 therapy were 8.4 months (95% CI, 7.7–9.2), and 9.8 months (95% CI, 8.3–11.4), respectively, in all studies combined.

In an analysis that considered time separately, the number of anti–PD-1–treated patients at risk of death was similar to that of the anti–PD-L1-treated group at 6 months (I^2^ = 91.2%; *P* = 0; OR, 1.65; 95% CI, 0.76–3.57; *P* = 0.204). At 12 months, the number of anti–PD-1–treated patients at risk of death was higher than that of anti–PD-L1–treated patients (I^2^ = 87.8%; *P* = 0; OR, 2.17; 95% CI, 1.04–4.44; *P* = 0.033).

However, the RMST values at the 12-month follow-up were 9.4 months (95% CI, 8.8–10.0) for anti–PD-1 therapy and 9.3 months (95% CI, 8.8–9.7) for anti–PD-L1 therapy (z = 0.26, *P* = 0.794).

**Figure 5:**
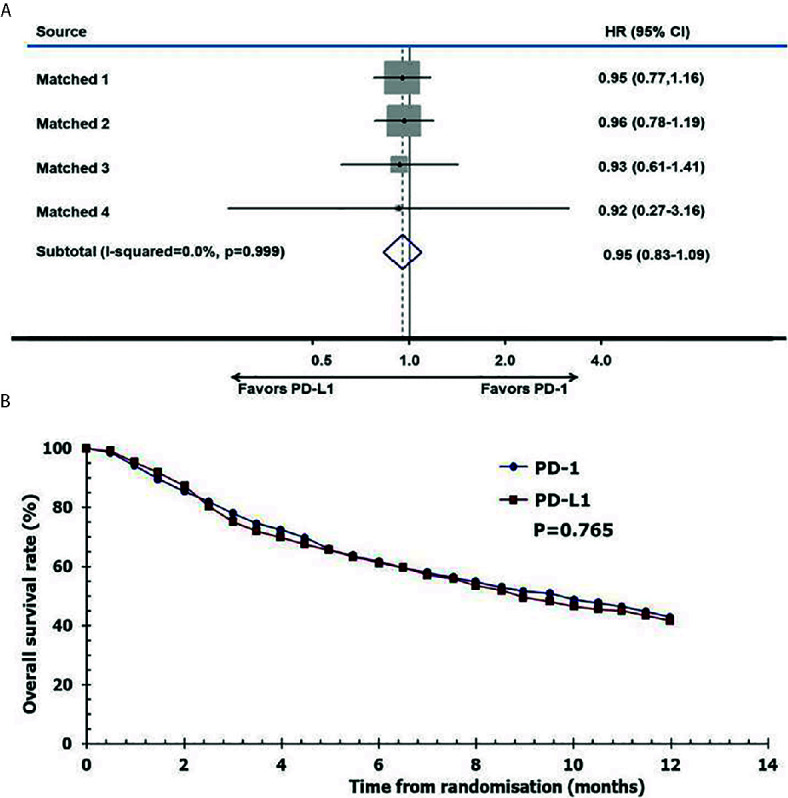
Pooled hazard ratio of survival. **(A)** meta-analysis of pooled hazard ratios of overall survival outcomes of anti–PD-1 versus anti–PD-L1 therapy, **(B)** overall survival of patients with immune checkpoint inhibitors using reconstructed survival data.

The reconstructed survival data were highly consistent with the published data. The Kaplan-Meier (KM) curves of patients in the anti–PD-1 and anti–PD-L1 groups were reconstructed at the common maximum follow-up time (12 months) for direct comparison (**Figure 5A**). No significant difference was observed between patients in the anti–PD-1 and anti–PD-L1 groups (12-month OS: 43% versus 42%, *P* = 0.765. I^2^ = 0; *P* = 0.999; HR, 0.95; 95% CI, 0.83–1.09; *P* = 0.474, **Figure 5B**).

## Discussion

In this study, we first used a well-designed mirror principle strategy that involved screening and pairing trial characteristics to minimize the potential bias in patients with platinum-resistant UC (20). The systematic review and meta-analysis was first adjusted for direct comparisons of anti–PD-1 and anti–PD-L1 therapy. The results suggested that anti–PD-1 therapy exhibited better antitumor activity than anti–PD-L1 therapy, with an acceptable safety profile.

Immune checkpoint inhibitors have remarkable clinical effects after progression with platinum-based chemotherapy (13, 33, 34). A meta-analysis showed that the ORR of immune-checkpoint inhibitors was 17.7% (*95% CI, 16%–20%*) (35). In another meta-analysis, the ORR of second-line or later treatment with immune checkpoint inhibitors was 18% (*95%* CI, 15%–22%) (8). Due to lack of head-to-head research or appropriate statistical methods, the difference in ORRs between patients treated with anti–PD-1 and anti–PD-L1 therapy has not been examined. Our results showed that the ORR of anti–PD-L1 therapy was 15% (95% CI, 15%–17%), and the ORR of anti–PD-1 therapy was 22% (95% CI, 18%–25%). Patient characteristics were matched using the mirror principle to minimize potential bias, and this method has been shown to be effective (20). With the mirror principle, the overall results showed that anti–PD-1 therapy exhibited better antitumor activity than anti–PD-L1 therapy in terms of the ORR and DCR.

The safety profiles of both therapies were acceptable. These immune checkpoint inhibitors were well tolerated in previous trials (13, 28, 31, 36). Grade 1–2 AEs were the most frequent treatment-related AEs and were manageable with expectant treatment (1, 15). In this analysis, the treatment-related AEs of anti–PD-1 therapy were comparable to those of anti–PD-L1 therapy. A network meta-analysis that included only two studies showed that pembrolizumab had advantages over atezolizumab in terms of serious AEs (9). However, we found that the incidence of treatment-related AEs and grade 3–5 treatment-related AEs associated with anti–PD-1 therapy were comparable to those of anti–PD-1 therapy.

In a previous study, immunotherapies had more obvious survival benefits than chemotherapy (9, 35). Even for monotherapy, immune checkpoint inhibitors also has delightful prognosis. The IMvigor130 trail provided evidence to support that anti–PD-L1 therapy plus chemotherapy can prolong the PFS of urothelial carcinoma patients (15). The Keynote-045 and IMvigor211 studies also demonstrated that the OS rate for pembrolizumab treatment was higher than that of chemotherapy in patients with platinum-resistant UC (11, 29). There were only two trials, which had insufficient power, that indicated no significant differences between PD-1 and PD-L1 blockade (19, 20). Using the same statistical methods, the median OS times with anti–PD-L/PD-L1 therapy were 8.4 months and 9.8 months, respectively, in all studies combined. At the common maximum follow-up time (12 months), although the rate of death of anti–PD-1–treated patients was lower, the OS of anti–PD-1–treated patients was comparable to that of anti–PD-L1–treated patients.

Differences exist in the mechanism of action between PD-1 and PD-L1 (16, 37, 38), which might explain the clinical differences in theory. PD-1 antibodies can bind to PD-1 to its ligands (PD-L1 and PD-L2), however, the interaction of PD-1 and PD-L2 remains intact, which may inhibit activation of T cells in PD-L1 antibodies (20). Therefore, the tumor might escape antitumor immune response through the PD-1/PD-L2 axis when being treated with anti–PD-L1, which may explain why patients receiving anti–PD-1 therapy had a better response rate than anti–PD-L1 therapy. Studies are ongoing and some patients had subsequent therapies that impact on survival outcomes. The results of this study may provide a reference for clinical studies that contributes to enhancing treatment awareness in patients with platinum-resistant UC.

Several limitations of this study should be noted. 1) Due to the lack of clinical trials, the trials included in this study were relatively limited after matching. However, this illustrates the importance and necessity of this research. 2) Because the details of all studies are not available, the study lacks individual patient data creates an important handicap when comparing and matching. The method used was an indirect comparison. However, the statistical methods were also confirmed (19, 20, 25). This study screened the research through the mirror matching method, which led to the inability of matching analysis of some cancer data. All analyses may be considered exploratory rather than hypothesis-tested in our study. 3) We could not distinguish the differences among various types of drugs. 4) Some information was incomplete. For example, AEs were classified into different systems instead of being analyzed individually. However, the main endpoint of ORR was a better comparison of the outcome than AEs in this study. For a few studies, a subgroup analysis of metastases or PD-L1 expression was not performed. We suggest that subgroup analyses including metastases or PD-L1 expression will provide information for future studies. 

## Conclusion

In summary, the results of our systematic comparison suggest that anti–PD-1 therapy exhibits better antitumor activity than PD-L1 therapy in patients with platinum-resistant UC. The safety profiles and survival outcomes for PD-1 and PD-L1 treatment were comparable. These findings may contribute to enhancing treatment awareness in patients with platinum-resistant UC treatment.

## Data Availability Statement

The raw data supporting the conclusions of this article will be made available by the authors, without undue reservation.

## Author Contributions

FZ and KX had full access to all the data in the study and take responsibility for the integrity of the data and the accuracy of the data analysis. Study conception and design: ZL, XL, HH, and FZ. Acquisition of data: ZL, XL, and HH. Analysis and interpretation of data: all authors. Drafting of the manuscript: ZL and XL. Critical revision of the manuscript for important intellectual content: all authors. Statistical analysis: ZL, XL, FZ, and HH. Obtaining funding: ZL. Administrative, technical, or material support: FZ. Supervision: FZ. All authors contributed to the article and approved the submitted version.

## Funding

This work was supported by the National Natural Science Foundation of China (Grant No. 81902610) and Science and Technology Planning Project of Shenzhen Municipality (CN) (JCYJ20190807145409328).

## Conflict of Interest

The authors declare that the research was conducted in the absence of any commercial or financial relationships that could be construed as a potential conflict of interest.

## Abbreviations

AE, adverse event; CI, confidence interval; CR, complete remission; DCR, disease control rate; HR, hazard ratio; OR, odds ratios; ORR, objective response rate; OS, overall survival; PD, progressive disease; PD-1/L1, programmed death 1/ligand 1; PFS, progression-free survival; RMST, restricted mean survival time; PR, partial response; SD, stable disease; UC, urothelial cancer.
